# Editorial: Genetic and immunological insights in solid tumors: comprehensive approaches to treatment

**DOI:** 10.3389/fgene.2026.1783167

**Published:** 2026-03-04

**Authors:** Yang Zhao, Cheng Ding, Zhi-jia Liu, Jing Wang, Lin Zhou

**Affiliations:** 1 Beijing Chaoyang Hospital, Capital Medical University, Beijing, China; 2 Eighth Medical Center of the General Hospital of the Chinese People’s Liberation Army, Beijing, China

**Keywords:** biomarker, immune assessment, immunotherapy, solid tumors, tumor immune microenvironment

The high heterogeneity and complex microenvironment of solid tumors have long been the core challenges in clinical treatment (Fu et al., 2021). Conventional therapeutic modalities, including surgery, radiotherapy and chemotherapy, often fail to achieve precise targeted therapy and maintain long - term efficacy (Vasan et al., 2019). With the progress of molecular biology and immunology, patient stratification based on genetic biomarkers, systematic analysis of the tumor immune microenvironment, and the construction of multi - dimensional prognostic models integrating clinical and molecular characteristics have gradually improved the survival benefits of tumor patients (Jiang et al., 2022). The above advancements on basic theories has not only deepened the understanding of the biological behaviors of solid malignant tumors, but also promoted and innovated the development of individualized cancer treatment strategies.

The Research Topic focuses on typical solid tumors such as pancreatic cancer and non-small cell lung cancer (NSCLC). From the perspectives of novel biomarker identification, construction of immune-related gene models, and analysis of real-world treatment patterns, these studies offer crucial evidence for the “genetic-immune integration” comprehensive treatment strategy and unveil the development trends and key issues in this field.

Accurate identification of biomarkers is a prerequisite for the stratified treatment of solid tumors, and biomarkers considering both genetic background and immune status exhibit higher clinical translatability. Ye et al.’s research on patients suffering from locally advanced pancreatic cancer ascertained that the Monocyte-to-High-Density Lipoprotein Ratio (MHR), serving as a novel immune - inflammatory - metabolic biomarker, is capable of independently predicting the Overall Survival (OS) and Disease - Free Survival (DFS) of patients subsequent to allogeneic vascular replacement pancreaticoduodenectomy. When MHR > 1.184, the 1-year mortality risk of patients increased significantly, with a median OS of only 12.0 months (lower than 27.0 months in the low MHR group). It jointly formed a prognostic evaluation system with preoperative creatinine and tumor TNM stage. This finding combines inflammatory responses (monocytes) and lipid metabolism (high-density lipoprotein), showing the close link between systemic immuno-metabolic imbalance and prognosis in pancreatic cancer patients, and offering a simple detection indicator for preoperative risk stratification and intervention target selection.

Zhao et al. has demonstrated that highly expression of PSMB6 in tumor tissues associated with poor prognosis. Mechanistically, PSMB6 regulates the epithelial-mesenchymal transition (EMT) process and immune cell infiltration, reduces the infiltration levels of anti-tumor immune cells such as CD8^+^ T cells and NK cells, and simultaneously upregulates the expression of the immune checkpoint molecule CD276, thereby promoting tumor immune escape. Knockdown of PSMB6 can significantly inhibit the proliferation, migration, and invasion of lung adenocarcinoma cells and induce their apoptosis. This study is the first to link proteasome dysfunction with tumor immune microenvironment remodeling, establishing the dual value of PSMB6 as a prognostic biomarker and potential therapeutic target for lung adenocarcinoma.

Zhao et al. found that inflammatory biomarkers such as the Neutrophil-to-Lymphocyte Ratio (NLR) and Monocyte-to-Lymphocyte Ratio (MLR) were associated with short-term survival but could not serve as independent prognostic factors for NSCLC patients; while traditional clinicopathological characteristics such as age, tumor stage, and differentiation degree still dominated long-term prognosis. This result suggests that the application of solid tumor biomarkers requires precise screening combined with tumor pathological subtypes to avoid confounding factor interference, and also emphasizes the supplementary value of inflammation-related indicators in short-term efficacy monitoring.

If a single biomarker represents a “point” breakthrough, multi-gene prognostic models achieve “full-scale” coverage, and real-world data provide key validation for the clinical translation of models and treatment strategies. Based on TCGA and GEO databases, Zhang et al. constructed a T cell-related prognostic model containing 16 genes (including LATS2, LDHA, and CXCL13) through Weighted Gene Co-expression Network Analysis (WGCNA) and LASSO Cox regression. The model exhibited stable prognostic predictive ability in both the training set and external validation set (1-, 3-, and 5-year AUC were 0.68, 0.72, and 0.69, respectively) and could effectively distinguish the differences in the immune microenvironment between high-risk and low-risk patients: the tumor microenvironment of high-risk patients displayed an immunosuppressive phenotype and was more sensitive to the MCL1 inhibitor AZD5991; low-risk patients had stronger immune activity and were more likely to benefit from IGF1R inhibitor treatment. This “gene model-risk stratification-treatment matching” pattern directly links genetic characteristics with drug sensitivity, providing an operable tool for personalized medication in NSCLC.

Real-world studies further shed light on the clinical status and gaps in biomarker-guided treatment. Kiiskinen et al.’s multi-country observational study on RET fusion-positive NSCLC revealed that only 31% of advanced NSCLC patients underwent RET fusion testing, with significant differences in detection rates among different countries (Taiwan 8% vs. United States 67%); although RET inhibitors have been approved for clinical use, the rate of targeted therapy in this cohort was only 16%, much lower than that of traditional chemotherapy (32%). This result reflects the bottlenecks in the clinical popularization of novel genetic biomarkers, including factors such as test accessibility, physician awareness, and drug accessibility, and also suggests that the translation “from laboratory to bedside” requires the collaborative promotion of policy support and clinical education. Meanwhile, the study confirmed that RET fusion-positive NSCLC patients had a higher proportion of never-smokers and adenocarcinoma subtypes, providing a reference for population characteristics in clinical screening.

In the future, the comprehensive treatment of solid tumors should move towards “multi-dimensional integration and full-cycle management”. In the research field, multi-center, prospective cohort studies need to be conducted to verify the general applicability of existing biomarkers and prognostic models; combined with emerging technologies such as single-cell sequencing and spatial transcriptomics, the spatiotemporal dynamic association between genetic variations and immune cell infiltration should be analyzed to explore more specific targets. Through the application of innovative immune assessment technologies, such as the MISS score, the body’s immune status is monitored across the entire treatment cycle.

Employing immune cell therapy and T-cell adjuvant therapy such as thymosin, addressing the issue of patients’ intolerance to radiotherapy or chemotherapy due to early - stage immune deficiency post - surgery, finally established a specialized tumor treatment system centered on immunology. In the clinical field, the accessibility of broad-spectrum detection technologies such as NGS should be improved, and a standardized process of “biomarker testing-risk stratification-treatment regimen matching” should be established; in the translational field, the linkage between basic research and clinical practice should be strengthened, and targeted clinical trials should be carried out to verify the efficacy of personalized regimens (Zhou et al., 2024; Zhu et al., 2024).

Currently, the treatment of solid tumors has gradually shifted from the traditional “one - size - fits - all” model to a comprehensive paradigm driven by both “genetic characteristics and immune status”. Paying attention to the improvement of the body’s immune status during the perioperative period contributes to enhancing the efficacy of comprehensive treatment. Through multi-angle exploration, the above five studies collectively outline the core direction of this field: taking novel biomarkers as the entry point, multi-gene models as analytical tools, and real-world data as support, ultimately achieving precise, personalized, and full-cycle treatment. Despite numerous remaining challenges, with the progress of research technologies and the improvement of clinical practice, the in-depth integration of genetics and immunology will surely bring dual benefits of longer survival and higher quality of life to solid tumor patients.
